# PGAM5-MAVS interaction regulates TBK1/ IRF3 dependent antiviral responses

**DOI:** 10.1038/s41598-020-65155-1

**Published:** 2020-05-20

**Authors:** Yu-qiang Yu, Marta Zielinska, Wei Li, Dominic B. Bernkopf, Christiane Silke Heilingloh, Markus F. Neurath, Christoph Becker

**Affiliations:** 1 Department of Medicine 1, Friedrich-Alexander-University, Erlangen, Germany; 2 Department of Biochemistry, Faculty of Medicine, Medical University of Lodz, Łódź, Poland; 3 College of Veterinary Medicine, Northeast Agricultural University, Harbin, China; 4 Experimental Medicine II, Nikolaus-Fiebiger-Center, Friedrich-Alexander University Erlangen-Nürnberg, Erlangen, Germany; 5 Department of Immune Modulation, Friedrich-Alexander-University, Erlangen, Germany; 6 Department of Infectious Diseases, University Hospital Essen, University of Duisburg-Essen, Essen, Germany

**Keywords:** Virology, Cell biology

## Abstract

Viral infections trigger host innate immune responses, characterized by the production of type-I interferons (IFN) including IFNβ. IFNβ induces cellular antiviral defense mechanisms and thereby contributes to pathogen clearance. Accumulating evidence suggests that mitochondria constitute a crucial platform for the induction of antiviral immunity. Here we demonstrate that the mitochondrial protein phosphoglycerate mutase family member 5 (PGAM5) is important for the antiviral cellular response. Following challenge of HeLa cells with the dsRNA-analog poly(I:C), PGAM5 oligomers and high levels of PGAM5 were found in mitochondrial aggregates. Using immunoprecipitation, a direct interaction of PGAM5 with the mitochondrial antiviral-signaling protein (MAVS) was demonstrated. In addition, PGAM5 deficient cells showed diminished expression of IFNβ and IFNβ target genes as compared to WT cells. Moreover, PGAM5 deficient mouse embryonic fibroblasts (MEFs) exhibited decreased phosphorylation levels of IRF3 and TBK1 when challenged with poly(I:C) intracellularly. Finally, PGAM5 deficient MEFs, upon infection with vesicular stomatitis virus (VSV), revealed diminished IFNβ expression and increased VSV replication. Collectively, our study highlights PGAM5 as an important regulator for IFNβ production mediated via the TBK1/IRF3 signaling pathway in response to viral infection.

## Introduction

The innate immune system is the first line of defense against an invasion of pathogens^1^. Several evolutionarily conserved pattern recognition receptors (PRRs) have been linked to innate immune defense, including Toll-like receptors (TLRs), RIG-I-like receptors (RLRs), Nod-like receptors (NLRs) and cytosolic DNA sensors. Different receptors interact with their corresponding ligands, thereby triggering various pathways to activate innate immune responses. RLRs such as RIG-I and melanoma differentiation associated protein 5 (MDA5) are major cytoplasmic viral RNA sensors^2^. Viral RNA activates RIG-I or MDA5 and transduces the signal through the mitochondrial antiviral-signaling protein (MAVS) which forms functional prion-like aggregates to activate downstream molecules such as TBK1^3–7^. TBK1 in turn activates IRF3 and induces the production of type I IFNs and other pro-inflammatory cytokines. The production of type I IFNs is a critical step in antiviral defense and adaptive immunity to clear pathogens^8^.

The mitochondrial phosphoglycerate mutase 5 (PGAM5) belongs to the PGAM family and has been implicated in a broad range of biological processes, for instance regulating certain cell death pathways, activating Wnt/β-catenin signaling and promoting the activation of the NLRP3 inflammasome^9–15^. Recently, PGAM5 multimerization has been identified as a key process to regulate mitochondrial fission and mitophagy^16^. However, the functional role of PGAM5 multimerization is still largely unknown. Thus, multimerization may be a general feature of PGAM5 in certain cell types or may occur as a consequence of stimulation of certain signaling pathways.

Interestingly, prion-like polymerization of MAVS has been shown to promote the activation of antiviral signaling pathways via activating type I IFN responses. It is currently unknown whether PGAM5 might be similarly involved as MAVS in regulating type-I IFN expression during antiviral responses.

Here we highlight that intracellular delivery of the dsRNA-analog poly(I:C) leads to the formation of PGAM5 multimers. In PGAM5 deficient HeLa cells and PGAM5 deficient murine embryonic fibroblasts (MEFs), we demonstrate decreased expression of IFNβ and IFNβ target genes following stimulation with intracellular poly(I:C), when compared to wild-type (WT) cells. Our analyses further revealed that PGAM5 regulates IFNβ expression via TBK1/IRF3 dependent pathways and directly interacts with MAVS. Finally, we provide evidence that PGAM5 is essential for the IFN pathway and antiviral response during vesicular stomatitis virus (VSV) infection. Taken together, we uncovered that PGAM5 regulates the antiviral response via TBK1/IRF3.

## Results

### Intracellular poly(I:C) induces the formation of PGAM5 multimers

**Figure 1:**
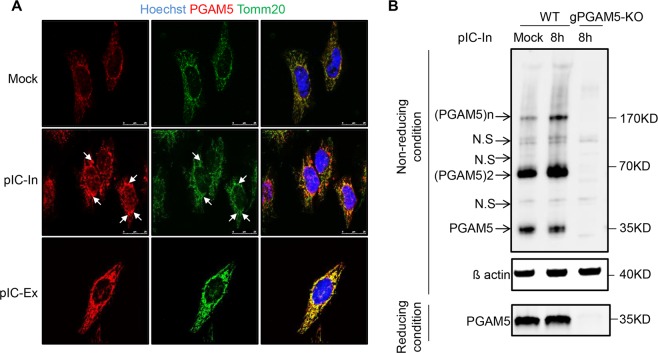
Intracellular poly(I:C) induces the formation of PGAM5 multimers. (**A**) Confocal microscopy imaging of HeLa cells stimulated with 50 µg/ml extracellular poly(I:C) (pIC-Ex) or 1 µg/ml intracellular poly(I:C) (pIC-In) for 8 h and stained with the following antibodies: anti-Tomm20 and anti-PGAM5. Hoechst was used to stain the nucleus. Extracellular poly(I:C) was added directly into the medium. Intracellular poly(I:C) was transfected into the cells. Arrowheads indicate PGAM5 aggregates. (**B**) SDS-PAGE analysis of PGAM5 multimers in lysates of HeLa cells under non-reducing or reducing conditions. HeLa cells were stimulated with 1 µg/ml intracellular poly(I:C) for 8 h. Cell lysates of PGAM5 CRISPR/Cas9 knockout cells were used as control to exclude non-specific bands. N.S, non-specific band.

To analyze the dynamics and location of PGAM5 after viral challenges, we used poly(I:C) to mimic RNA virus infection in HeLa cells. Cells were stained for PGAM5 to analyze location and expression. Confocal fluorescence microscopy revealed that PGAM5 staining overlapped with the mitochondrial marker Tomm20 in both mock treated and poly(I:C) transfected HeLa cells (Fig. 1A), suggesting that PGAM5 remains located in the mitochondria following stimulation. Strikingly, PGAM5 appeared to localize to aggregates in response to intracellular poly(I:C) stimulation (arrowhead, Fig. 1A). Although extracellular poly(I:C) treatment activated the TLR3 pathway, as indicated by rapid induction of phospho-STAT1 in HeLa cells, we could not detect PGAM5 aggregates (Figs. S1A, 1A), suggesting that PGAM5 forms multimers, specifically in cells challenged with intracellular RNA.

To further address the formation of PGAM5 multimers, intracellular poly(I:C) treated HeLa cells were lysed in non-reducing conditions and analyzed by SDS-PAGE (Fig. 1B). We were able to detect several specific bands with a molecular weight of 32 kDa or multiples thereof, indicating that PGAM5 exists not only as a monomer, but also as dimers ((PGAM5)2) and PGAM5 multimers ((PGAM5) n). Interestingly, PGAM5 multimers, although present at the steady state, significantly increased after intracellular poly(I:C) treatment. Of note, total PGAM5 protein levels of samples which were lysed in RIPA buffer and separated by SDS-PAGE remained constant before and after intracellular poly(I:C) treatment. β-mercaptoethanol (BME) treatment partly reduced PGAM5 multimerization (Fig. S1B), suggesting that PGAM5 multimer formation may rely on disulfide bonds. Collectively, our data indicated that the presence of intracellular RNA led to PGAM5 multimer formation and localization at mitochondrial aggregates.

### PGAM5 regulates intracellular poly(I:C)-induced IFNβ expression

**Figure 2:**
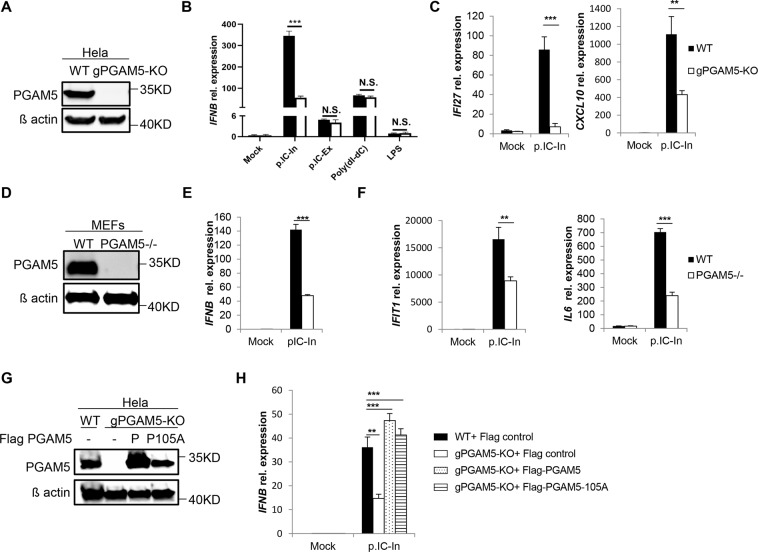
PGAM5 regulates intracellular poly(I:C)-induced IFNβ expression. (**A**) Immunoblot analysis of PGAM5 in lysates of WT and PGAM5 CRISPR/Cas9 knockout HeLa cells. β-actin served as a loading control. (**B**) mRNA expression of *IFNB* in WT and PGAM5 CRISPR/Cas9 knockout HeLa cells treated with vehicle (Mock), 1 µg/ml intracellular poly(I:C) (p.IC-In), 50 µg/ml extracellular poly(I:C) (p.IC-Ex), 1 µg/ml intracellular poly(dI:dC), or 100 ng/ml LPS. N.S., not significant. (**C**) mRNA expression of *IFI27* and *CXCL10* in WT and PGAM5 CRISPR/Cas9 knockout HeLa cells stimulated with 1 µg/ml intracellular poly(I:C) for 8 h. (**D**) Immunoblot analysis of PGAM5 in lysates of WT and PGAM5−/− Mouse Embryonic Fibroblasts (MEFs). β-actin served as a loading control. (**E,F**) mRNA expression of *Ifnb, Ifit1* and *Il6* in WT and PGAM5−/− MEFs stimulated with 1 µg/ml intracellular poly(I:C) for 8 h. (**G**) Immunoblot of ectopic PGAM5 expression in lysates of PGAM5 CRISPR/Cas9 knockout HeLa cells transfected for 48 h with full-length PGAM5 (P) vectors or phosphatase mutant PGAM5 (P105A) vectors. Lysates from empty vector transfected WT and PGAM5 CRISPR/Cas9 knockout HeLa cells were used as control. (**H**) mRNA expression of *IFNB* in corresponding HeLa cells stimulated with or without intracellular poly(I:C) for 8 h. WT + Flag, WT cells transfected with empty vector. gPGAM5-KO + Flag, PGAM5 CRISPR/Cas9 knockout cells transfected with empty vector. gPGAM5-KO + Flag PGAM5, PGAM5 CRISPR/Cas9 knockout cells transfected with full-length PGAM5 vectors. gPGAM5-KO + Flag PGAM5-105A, PGAM5 CRISPR/Cas9 knockout cells transfected with phosphatase mutant PGAM5 vectors. Experiments were performed three times and representative data are shown. Data are presented as mean +SD and student’s t-test was used for statistical calculation. **P < 0.01 and ***P < 0.001.

To elucidate a functional contribution of PGAM5 during antiviral responses induced by intracellular poly(I:C), PGAM5 deficient HeLa cells were generated using the CRISPR/Cas9 technology (Fig. 2A). Together with wild type (WT) cells, PGAM5 knockout HeLa cells were challenged with multiple ligands including intracellular/extracellular poly(I:C), poly(dI:dC) and lipopolysaccharide (LPS). As expected, *IFNB* expression was significantly induced by these ligands in WT cells. Interestingly, PGAM5 deficiency specifically attenuated *IFNB* expression induced by intracellular poly(I:C) but not when poly(I:C) was simply added into medium (Fig. 2B), suggesting that intracellular RNA sensors rather than membrane-bound TLR3 require PGAM5. In line with the diminished *IFNB* expression induced by PGAM5 deficiency, loss of PGAM5 decreased the expression of *IFI27* and *CXCL10*, two downstream genes of IFNβ signaling in HeLa cells (Fig. 2C). In order to further investigate whether this observation is a HeLa cell specific effect of PGAM5, we isolated murine embryonic fibroblasts (MEFs) from WT and PGAM5 −/− mice (Fig. 2D). Similar to the results obtained from the experiments performed with the HeLa cell line, PGAM5 deficient MEFs showed a significantly decreased expression of *Ifnb* and IFNβ downstream genes following stimulation with intracellular poly(I:C) but not when poly(I:C) was simply added into the medium (Fig. 2E, F, S2A). Intracellular poly(I:C) has been identified to activate the RIG-I like receptors^17^. In line with this finding, the loss of PGAM5 also impaired *Ifnb* expression induced by the RNA mimic 5´pppdsRNA (Fig. S2B), a specific synthetic ligand that activates the RIG-I pathway^18^.

To assess the possibility that attenuated IFNβ expression might occur due to off-target effects of PGAM5 gRNAs, we reconstituted PGAM5 expression in PGAM5 knockout HeLa cells. The exogenously induced PGAM5 expression was verified by Western Blot analyses (Fig. 2G). Overexpression of PGAM5 restored *IFNB* expression caused by PGAM5 deficiency in intracellular poly(I:C) treated HeLa cells (Fig. 2H). Considering that PGAM5 has been identified as a phosphatase^12^, the phosphatase domain was mutated to generate a phosphatase-dead PGAM5 expression vector (PGAM5-105A). PGAM5-105A vector was transfected into PGAM5 knockout cells and compared to HeLa cells reconstituted with wild type. Interestingly, restored *IFNB* expression was observed under both experimental conditions.

PGAM5 has two forms: a long form (PGAM5L) and a short form (PGAM5S), which are generated from alternative splicing within the C domain^12^. In order to study whether high level of IFNβ expression relies on the C domain, we expressed the short and long PGAM5 forms in PGAM5 knockout HeLa cells (Fig. S2C). Restored *IFNB* expression was observed in both cells (Fig. S2D), suggesting that the C domain is not essential for PGAM5-dependent IFNβ expression. In summary, PGAM5 functions as a mediator of intracellular RNA induced IFNβ response and its function is independent of its phosphatase activity.

### PGAM5 interacts with MAVS and regulates TBK1/IRF3 dependent IFNβ expression

**Figure 3:**
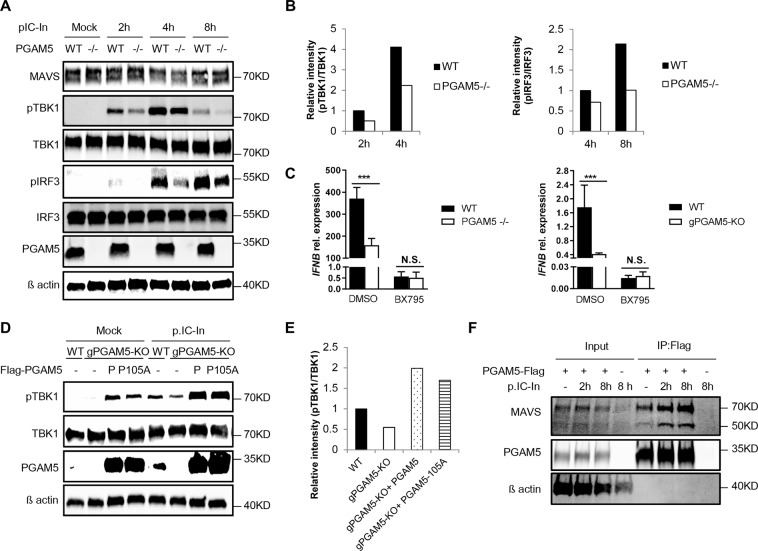
PGAM5 interacts with MAVS by regulating TBK1/IRF3 dependent IFNβ expression. (**A**) Immunoblot of phosphorylated or total proteins in lysates of WT and PGAM5−/− MEFs stimulated with intracellular poly(I:C) at different time points. (**B**) Relative analysis of pTBK1 (left panel) and pIRF3 (right panel) intensity. The total protein level of IRF3 or TBK1 was used for normalization. (**C**) mRNA expression of *Ifnb* in MEFs (left panel) or *IFNB* in HeLa cells (right panel) stimulated with intracellular poly(I:C) in the presence or absence of TBK1 inhibitor BX795. DMSO was used as control treatment. Experiments were performed three times and representative data are shown. Data are presented as mean +SD and student’s t-test was used for statistical calculation. ***P < 0.001. N.S., not significant. (**D**) Immunoblot from lysates of PGAM5 CRISPR/Cas9 knockout HeLa cells transfected for 48 h with WT PGAM5 vectors or phosphatase-mutant PGAM5 vectors. Lysates from empty vector transfected WT and PGAM5 CRISPR/Cas9 knockout HeLa cells were used as control. (**E**) Relative analysis of pTBK1 intensity (from D). The total protein level of TBK1 was used for normalization. (**F**) Immunoprecipitation and immunoblot of PGAM5 CRISPR/Cas9 knockout HeLa cells transfected for 48 h with or without full-length PGAM5 vectors followed by intracellular poly(I:C) stimulation.

IFNβ production induced by intracellular poly(I:C) depends on the activation of the RIG-I signaling pathway^17^. To investigate the effect of PGAM5 on RIG-I signaling, key molecules of the underlying pathway including MAVS, TBK1 and IRF3 were analyzed in MEFs challenged with intracellular poly(I:C) at various time points. As expected, intracellular poly(I:C) treatment induced rapid phosphorylation of IRF3 and TBK1 in WT MEFs. In contrast, PGAM5 deficient MEFs showed impaired phosphorylation of IRF3 and TBK1 (Fig. 3A,B). In order to functionally test whether PGAM5 regulates IFNβ production via a TBK1/IRF3 dependent pathway, we analyzed the *Ifnb* expression in the presence of a TBK1-specific inhibitor: BX795. In the presence of BX795, *IFNB* expression in response to intracellular poly(I:C) treatment was greatly diminished in WT MEFs (Fig. 3C, left panel). Under the same experimental conditions, no further *Ifnb* decrease was observed in PGAM5 deficient MEFs, suggesting that PGAM5 acts via a TBK1 dependent pathway. Similar results were obtained when the experiments were repeated in HeLa cells (Fig. 3C, right panel). In order to study whether PGAM5 overexpression might drive TBK1 phosphorylation, we next analyzed the phosphorylation levels of TBK1 in PGAM5-reconstituted HeLa cells (Fig. 3D,E). Again, PGAM5 deficiency impaired phosphorylation of TBK1 in response to intracellular poly(I:C) treatment. Overexpression of PGAM5 restored the diminished TBK1 phosphorylation caused by PGAM5 deficiency. Interestingly, rapid induction of phospho-TBK1 was observed in HeLa cells overexpressing either WT or phosphatase-dead PGAM5, even in the absence of intracellular poly(I:C) treatment. Taken together, our experiments suggest that PGAM5 functions upstream of TBK1 and that high-level expression of PGAM5 can be sufficient to induce TBK1 phosphorylation independent of its phosphatase activity.

In order to better understand the role of PGAM5 in the signaling pathway upstream of TBK1/IFR3, we next focused on MAVS. MAVS and PGAM5 are both located on the mitochondrial membrane and MAVS has been shown to be vital for activation of TBK1/IRF3 responses via forming functional aggregates^5,7,14,19^. Initially, we performed a semi-denaturing detergent agarose-gel electrophoresis (SDD-AGE) assay to detect MAVS aggregates on cells stimulated with intracellular poly(I:C) (Fig. S3A). As expected, intracellular poly(I:C) treatment induced the formation of MAVS aggregates. Of note, we did not detect differences in MAVS aggregation between WT and PGAM5 −/− cells, suggesting that PGAM5 promotes interferon-expression without directly regulating MAVS aggregation. In another set of experiments, we generated MAVS knockout HeLa cells to test the effect of MAVS deficiency on PGAM5 multimerization (Fig. S3B). Diminished *IFNB* expression in MAVS knockout HeLa cells confirmed the key role of MAVS in the RIG-I pathway. However, MAVS deficiency did not block PGAM5 multimerization (Fig. S3C), indicating that the formation of PGAM5 multimers and MAVS aggregates are independent of each other.

Given the above findings, we next studied whether PGAM5 and MAVS interact upon stimulation with intracellular poly(I:C). Although PGAM5 deficiency did not impact the protein levels of MAVS during intracellular poly(I:C) stimulation (Fig. 3A), using immunoprecipitation, we could demonstrate a physical interaction between PGAM5 and MAVS. Of note, the interaction of PGAM5 and MAVS was significantly enhanced by intracellular poly(I:C) treatment (Fig. 3F), suggesting that PGAM5 promotes the antiviral response by direct effects on MAVS. Taken together, these data demonstrated that PGAM5 interacts with MAVS and regulates TBK1/IRF3 dependent IFNβ expression during intracellular RNA treatment.

### PGAM5 regulates VSV-induced IFNβ expression and inhibits VSV replication

**Figure 4:**
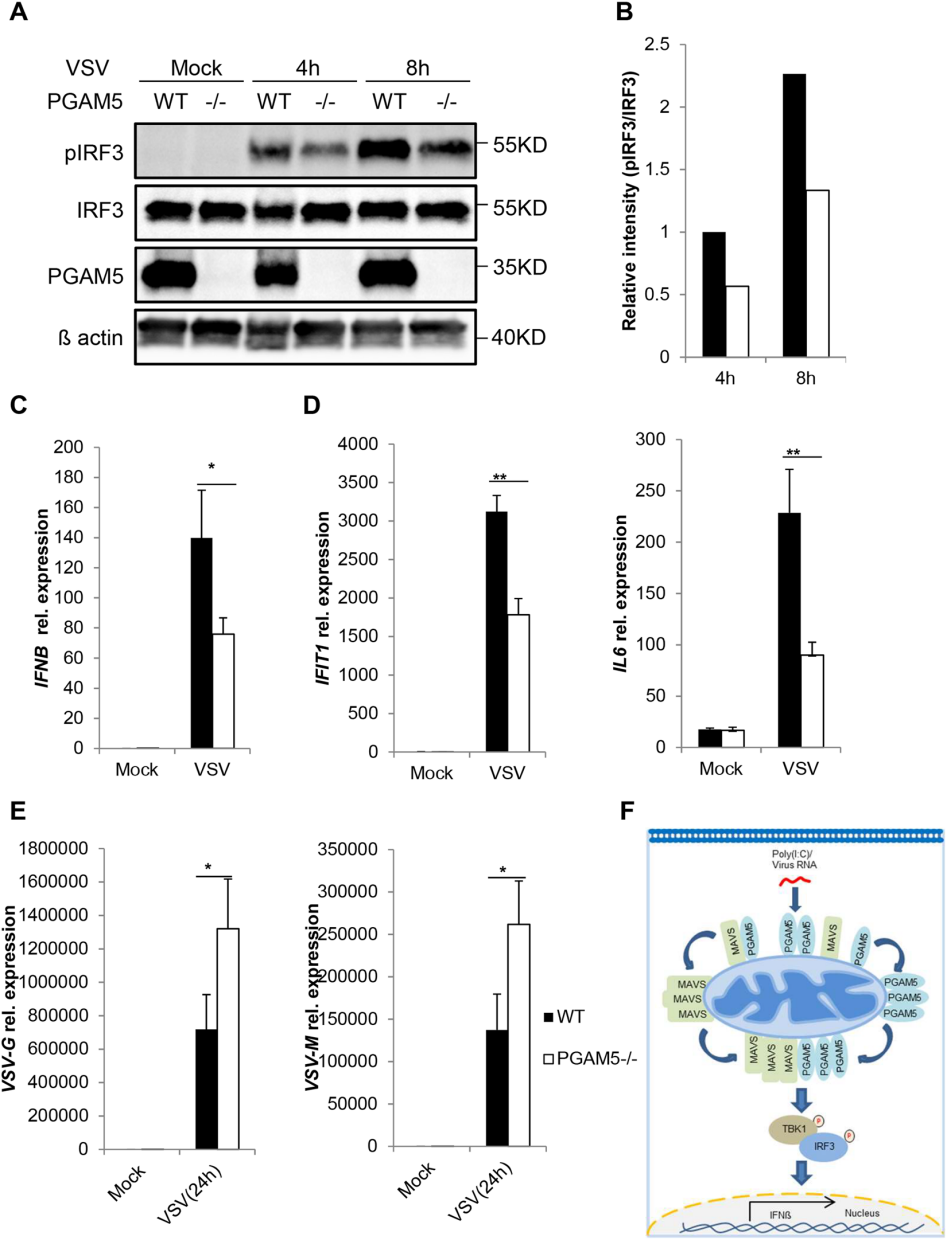
PGAM5 regulates vesicular stomatitis virus (VSV)-induced IFNβ expression and inhibits VSV replication in MEFs. (**A**) Immunoblot of phosphorylated or total proteins in lysates of WT and PGAM5−/− MEFs infected with VSV for indicated hours. (**B**) Relative analysis of pIRF3 intensity as shown in (**A**). The total protein level of IRF3 was used for normalization. (**C,D**) mRNA expression of *Ifnb, Ifit1* and *Il6* in WT and PGAM5−/− MEFs infected with VSV. Experiments were performed three times and representative data are shown. Data are presented as mean +SD and student’s t-test was used for statistical calculation. *P < 0.05 and **P < 0.01. (**E**) qPCR analysis of *VSV-G* and *VSV-M* in WT and PGAM5−/− MEFs infected with VSV. *P < 0.05. (**F**) A proposed antiviral signaling pathway related to PGAM5.

Poly(I:C) is a well-established ligand and widely used in mimicking RNA virus infection and viral induced IFN expression. Given the fact that PGAM5 is required for intracellular poly(I:C) induced responses, we finally examined whether PGAM5 may regulate the cellular immune defense to vesicular stomatitis virus (VSV), a virus known to trigger RIG-1 signaling via intracellular viral RNA. WT MEFs exposed to live VSV demonstrated pronounced phosphorylation of IRF3 (Fig. 4A,B). In contrast, PGAM5 deficient MEFs exposed to VSV under the same experimental condition showed a diminished phosphorylation of IRF3. In line with this finding, VSV induced *Ifnb, Ifit1* and *Il6* mRNA levels were decreased in PGAM5 deficient MEFs as compared to controls (Fig. 4C,D). Finally, in order to study whether PGAM5 would have an impact on the replication of VSV, we determined the viral load at 24 h post infection (Fig. 4E). Indeed, PGAM5 deficient MEFs showed a significantly higher virus load as compared to WT MEFs exposed to VSV, suggesting that PGAM5 is an important regulator of antiviral immunity. Collectively, our results delineate a signaling pathway in which PGAM5 aggregates with MAVS to regulate TBK1/IRF3 dependent IFNβ expression and antiviral responses (Fig. 4F).

## Discussion

As a mitochondrial molecule, PGAM5 has been reported to play critical roles in cell death, mitochondrial biogenesis and inflammation^9–15^. Although, previous studies reported that PGAM5 promotes inflammasome activation^15^, the functional role of PGAM5 in antiviral immune responses is largely unknown. Here we revealed the formation of PGAM5 multimers upon intracellular sensing of RNA and identified PGAM5 as a key molecule of antiviral immunity by regulating IFNβ expression. At a molecular level, we further unraveled that PGAM5 physically interacts with MAVS and regulates IFNβ expression via TBK1/IRF3 dependent pathways. Finally, VSV was used to infect MEFs and the loss of PGAM5 decreased IFNβ responses and promoted VSV replication.

Our results clearly demonstrated that intracellular poly(I:C) induced PGAM5 multimers formation. Structural and biochemical data have suggested that purified PGAM5 itself can form multimers and endogenous PGAM5 forms multimers while challenged with CCCP or NLRP3 inflammasome agonist^15,20,21^. Using high concentrations of BME, which was able to eliminate MAVS aggregates^7^, we showed that PGAM5 multimer levels decreased but did not disappear. Of note, treatment with BME did not affect the poly(I:C)-induced upregulation of PGAM5 multimerization. Since BME is a reducing agent that was used to destroy disulfide bonds, the PGAM5 multimers may only partially contain disulfide bonds. It will be interesting to determine the precise biochemical nature of PGAM5 multimers in future investigations.

Previous studies demonstrated that PGAM5 regulates CCCP-induced mitophagy and promotes NLRP3 inflammasome activation. Interestingly, CCCP and NLRP3 inflammasome agonists also induced the formation of endogenous PGAM5 multimers in living cells^15,20,22^, suggesting the strong connection between PGAM5 multimer formation and the functional activity of PGAM5 in multiple pathways. Double-stranded RNA, which is present in many RNA viruses, induces robust immune responses via pattern recognition receptors, such as Toll-like receptor 3 (TLR3), and RIG-I. TLR3 is expressed either on the cell surface or in intracellular endosome compartments, while RIG-I is expressed in the cytosol. TLR3 preferentially recognizes viral RNA presented extracellularly while RIG-I expression in the cytoplasm allows the detection of actively replicating viruses^23,24^. In our experiments, PGAM5 promoted IFNβ expression only if poly(I:C) was delivered into the cells by transfection, suggesting that PGAM5 is involved in RIG-I mediated signaling. RIG-I has recently been demonstrated as an essential molecule in VSV-induced cytokine production^25^. Indeed, when PGAM5-deficient cells were exposed to VSV, IFNβ-induction was diminished when compared to WT cells, further supporting our conclusions. Given that intracellular poly(I:C) activates RIG-I and transduces the signal through MAVS-TBK1-IRF3 to produce IFNβ, we conclude that PGAM5 regulates IFNβ production via a TBK1/IRF3 dependent cytoplasmatic pathway. Interestingly, we observed that PGAM5 overexpression can be sufficient to induce TBK1 phosphorylation. This finding suggests that PGAM5 functions upstream of TBK1 and that PGAM5 levels within the cell can affect the activation level of TBK1. MAVS is a key regulator for activating antiviral innate immune responses via the formation of functional aggregates. Unlike PGAM5 multimers that have been identified in the steady state, MAVS aggregates were only detected after intracellular poly(I:C) stimulation. Our data showed that the formation of PGAM5 multimers and MAVS aggregates are independent of each other, suggesting that PGAM5 may modify MAVS independently of the formation of aggregates. We further uncovered that PGAM5 and MAVS physically interact and this interaction was significantly enhanced by intracellular poly(I:C) stimulation. Given the crucial role of MAVS in this pathway, our data suggest that PGAM5 promotes the antiviral response by direct effects on MAVS.

In summary, our data highlight the formation of PGAM5 multimers and the importance of PGAM5 for regulating IFNβ production via TBK1/IRF3 dependent pathway. One may speculate that PGAM5 serves as a scaffolding protein to allow formation of a PGAM5-MAVS multimeric complex which is essential for efficient IRF3 and TBK1 activation.

## Materials and Methods

### Cells

MEFs were isolated from day 13.5 embryos and cultured in complete Dulbecco’s modified Eagle’s medium (DMEM) (Gibco) supplemented with 10% fetal bovine serum, penicillin (100 U/ml), and streptomycin (100 μg/ml). HeLa cells were maintained in the some medium as MEFs.

To stimulate cells with intracellular RNA, 1 µg/ml low molecular weight poly(I:C) (Invivogen) or 0.5 µg/ml 5′pppdsRNA (Invivogen) was transfected into cells by using Lyovec (Invivogen) or Lipofectamine 2000 (Invitrogen) according to the manufacturers instructions. To stimulate cells with intracellular DNA, 1 µg/ml Poly(dI:dC) (Invivogen) was transfected into cells by using Lipofectamine 2000 (Invitrogen). To active TLR3 or TLR4, cells were incubated with 50 µg/ml low molecular weight poly(I:C) (Invivogen) or 100 ng/ml LPS (Sigma).

For virus infection, VSV (Indiana strain; kindly provided by Prof. Peter Stäheli; Universitätsklinikum Freiburg) was propagated on BHK21 cells. MEFs were infected with VSV (0.1 MOI) in FBS-free medium in the incubator for 1 h and then switched to complete medium containing 10% FBS.

### Generation of HeLa knockout cells

For generation of PGAM5 and MAVS knockout cells from single-cell colonies, PGAM5 CRISPR/Cas9 KO Plasmid (Santa Cruz) or MAVS CRISPR/Cas9 KO Plasmid (Santa Cruz) was transfected into HeLa cells. GFP-positive cells were sorted into 96-well plates (single cell/well) by flow cytometry (FACSAria II; BD). Sufficiency of knockout was verified by immunofluorescence and Western Blot (WB) analysis.

### RNA extraction and real-time quantitative PCR

Total RNA was extracted from cultured cells using peqGOLD Total RNA Kit (Peqlab, Erlangen, Germany) according to the manufacturer’s protocol and reverse transcribed into cDNA by the SCRIPT cDNA Synthesis Kit (Jena Bioscience). Quantitative real-time PCR was carried out using specific primers targeting VSV and QuantiTect Primer assays (Qiagen) for other genes. Primers targeting VSV-G or VSV-M (forward/reverse; 5′ to 3′’) are as follows:

VSV-G: CAAGTCAAAATGCCCAAGAGTCACA/TTTCCTTGCATTGTTCTACAGATGG

VSV-M: TATGATCCGAATCAATTAAGATATG/GGACGTTTCCCTGCCATTCCG ATG

The mRNA expression of the reference gene hypoxanthine guanine phosphoribosyl transferase (*HPRT*) was used to normalize cDNA levels.

### Immunofluorescence

Cells grown on 8-well culture slides (Falcon) were fixed with 4% paraformaldehyde, permeabilized with 0.1% Triton X-100 and then incubated with indicated antibodies. Nuclei were stained with Hoechst (Sigma-Aldrich). Imaging of the cells was carried out using a Leica laser-scanning confocal microscope.

### Immunoprecipitation (IP) assay

For immunoprecipitation, whole-cell extracts were prepared in the presence of native lysis buffer [20 mM Tris-HCl (PH 7.5), 100 mM NaCl, 10% glycerol, 0.5% NP-40, and 0.5% DDM] supplemented with protease inhibitors (Complete Mini Protease Inhibitor Cocktail, Roche) and phosphatase inhibitors (PhosphoStop Phosphatase Inhibitor Cocktail, Roche). Cell lysates were centrifuged for 20 min at 19,000 g and supernatants were collected and incubated with anti-Flag (M2) agarose for 1 h at room temperature. The agarose beads were washed five times with native lysis buffer and co-precipitated proteins were eluted using SDS–PAGE loading buffer.

### Western blot (WB) analysis

For immunoblot analysis of lysates under non-reducing conditions, whole-cell extracts were prepared in native lysis buffer as motioned above. Supernatant proteins from cell lysates were quantified and supplemented with native sample buffer (Bio-Rad). Proteins were separated by SDS-PAGE using a MiniProtean-TGX gel (4–15% polyacrylamide; Bio-Rad) and blotted on nitrocellulose membrane (Whatman) followed by incubation with indicated antibodies.

For immunoblot analysis of lysates under reducing conditions, whole-cell extracts were prepared in RIPA lysis buffer (Pierce) supplemented with protease and phosphatase inhibitors. Supernatant proteins from cell lysates were supplemented with LDS Sample Buffer (ThermoFisher) and heated under 95 °C for 5 min and then used for SDS-PAGE assay.

Anti-human PGAM5 antibody was obtained from Sigma, anti-mouse PGAM5 antibody was obtained from Santa Cruz Biotechnology, anti-pIRF3, anti-TBK1, anti-pTBK1 and anti-MAVS were obtained from Cell Signaling Technology, anti-mouse IRF3 and anti-β-actin antibodies were obtained from Abcam.

### Semi-denaturing detergent agarose-gel electrophoresis (SDD-AGE) assay

SDD-AGE was performed as previously published^7^. In brief, crude mitochondria were isolated from MEFs and suspended in sample buffer (0.5 x TBE, 10% glycerol, 2% SDS, and 0.0025% bromophenol blue) and loaded onto a vertical 1.5% agarose gel. After electrophoresis for 40 min in running buffer (1 × TBE and 0.1% SDS) with a constant voltage of 100 V at 4 °C, western blotting was performed.

### Statistical analysis

Significances were determined using the two-tailed student’s t-test. Differences were considered significant at *p ≤ 0.05; **p ≤ 0.01; ***p ≤ 0.001; N.S. = not significant (p > 0.05).

## Supplementary information

Supplementary Information.
