# Supplementary material for: PGAM5-MAVS interaction regulates TBK1/ IRF3 dependent antiviral responses
Source: Sci Rep. 2020 May 20;10:8323. doi: 10.1038/s41598-020-65155-1 (PMC7239892; doi:10.1038/s41598-020-65155-1)
Supplement: Supplementary file 1 — Supplementary Information. [file 41598_2020_65155_MOESM1_ESM.pdf]

Supplementary Information

PGAM5-MAVS interaction regulates TBK1/ IRF3 dependent antiviral responses

Yu-qiang Yu[1], Marta Zielinska[1, 2], Wei Li[3], Dominic B. Bernkopf[4], Christiane Silke Heilingloh[5, 6], Markus F. Neurath[1], Christoph Becker[1*]

[1] Department of Medicine 1, Friedrich-Alexander-University, Erlangen, Germany

[2] Department of Biochemistry, Faculty of Medicine, Medical University of Lodz, Łódź, Poland.

[3] College of Veterinary Medicine, Northeast Agricultural University, Harbin, China

[4] Experimental Medicine II, Nikolaus-Fiebiger-Center, Friedrich-Alexander University Erlangen-Nürnberg, Erlangen, Germany.

[5] Department of Immune Modulation, Friedrich-Alexander-University, Erlangen, Germany.

[6] Department of Infectious Diseases, University Hospital Essen, University of Duisburg-Essen, Essen Germany

Address correspondence to:

Christoph Becker, PhD

Department of Medicine 1,

Friedrich-Alexander-University Erlangen-Nuremberg

Erlangen, Germany

Phone: (0049) 9131-85 35 886

Email : christoph.becker@uk-erlangen.de

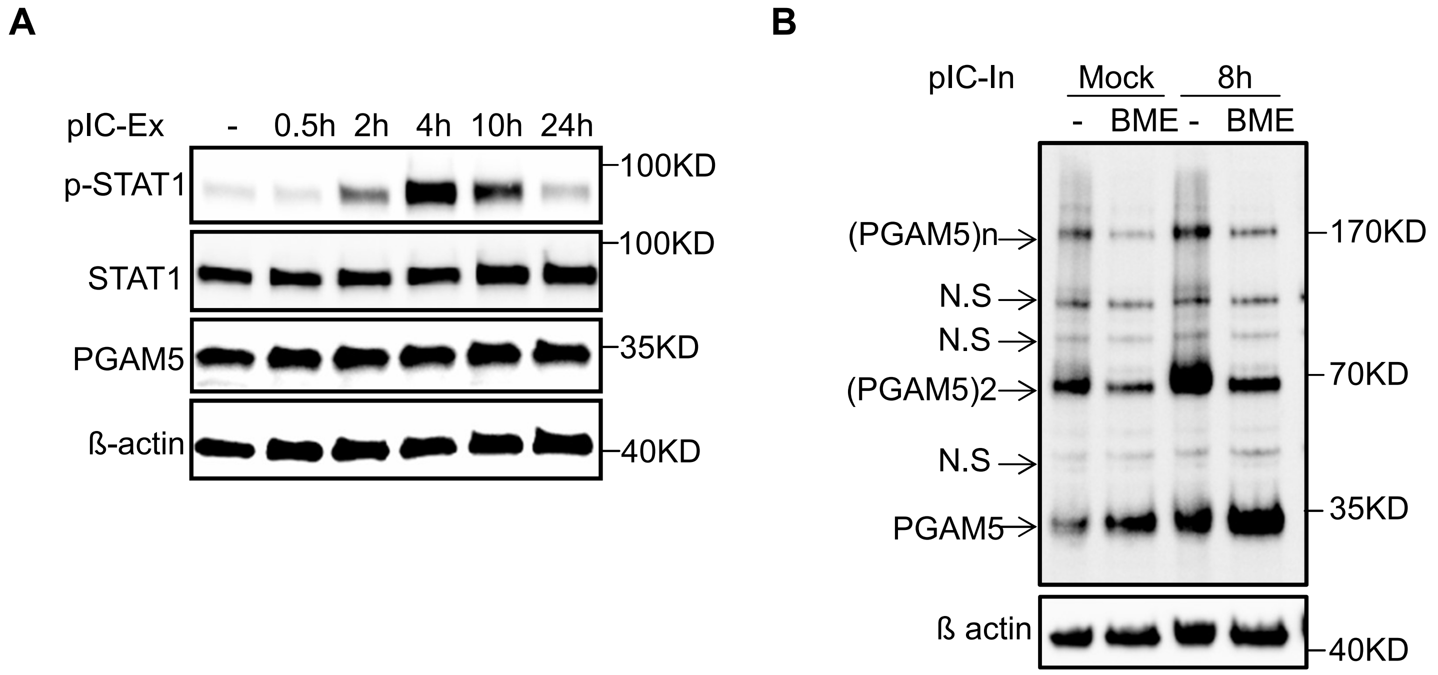
A
pIC-Ex
-
0.5h
2h
4h
10h
24h
p-STAT1
100KD
STAT1
100KD
PGAM5
35KD
ß-actin
40KD
B
pIC-In
Mock
8h
-
BME
-
BME
(PGAM5)n
N.S
N.S
(PGAM5)2
N.S
PGAM5
170KD
70KD
35KD
ß actin
40KD

S Figure 1

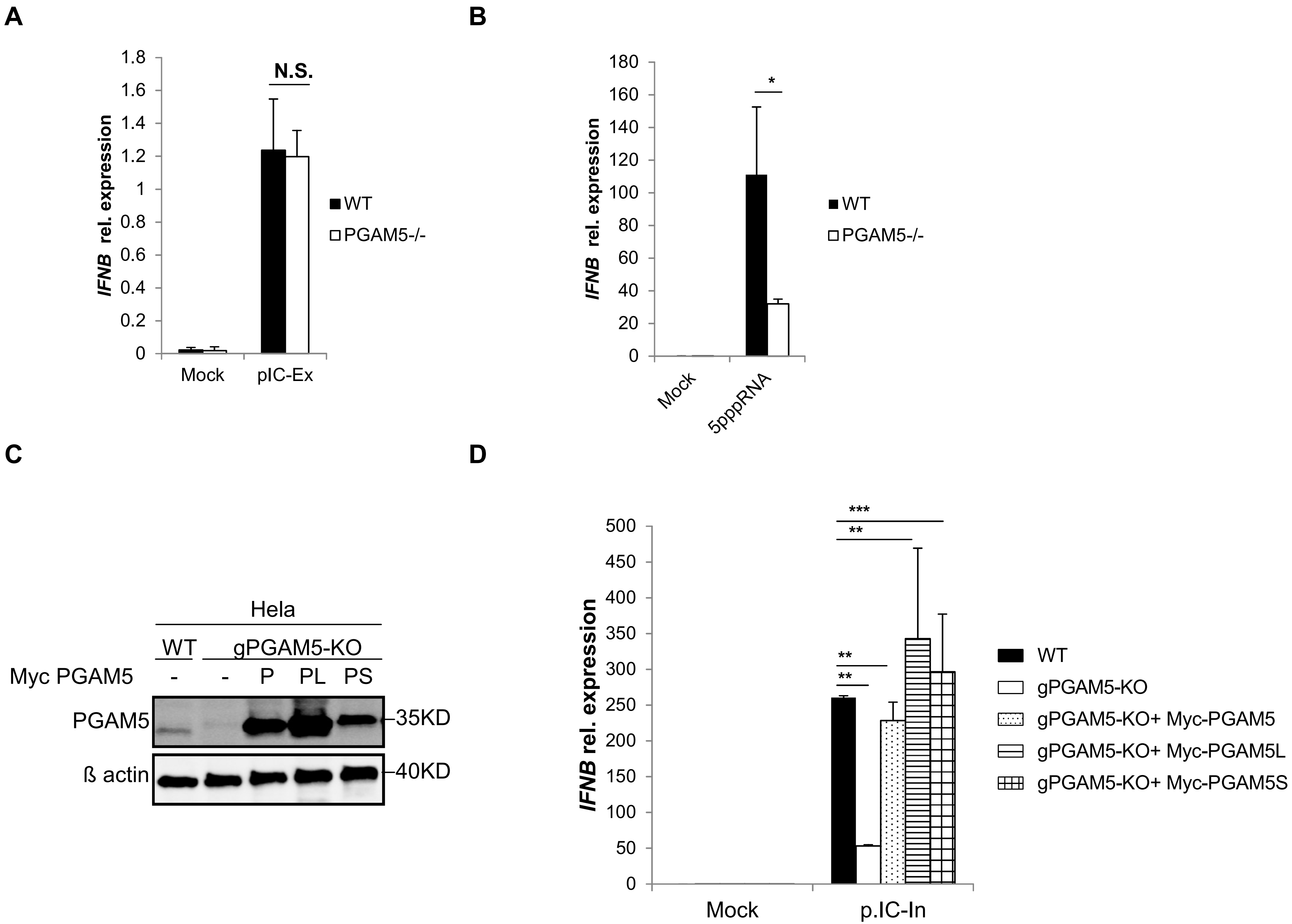
A
1.8
1.6
1.4
1.2
1
0.8
0.6
0.4
0.2
0
N.S.
IFNB rel. expression
WT
PGAM5-/-
Mock
pIC-Ex
B
180
160
140
120
100
80
60
40
20
0
*
IFNB rel. expression
WT
PGAM5-/-
Mock
5pppRNA
C
Hela
WT
gPGAM5-KO
Myc PGAM5
-
-
P
PL
PS
PGAM5
35KD
ß actin
40KD
D
500
450
400
350
300
250
200
150
100
50
0
***
**
**
**
IFNB rel. expression
WT
gPGAM5-KO
gPGAM5-KO+ Myc-PGAM5
gPGAM5-KO+ Myc-PGAM5L
gPGAM5-KO+ Myc-PGAM5S
Mock
p.IC-In

S Figure 2

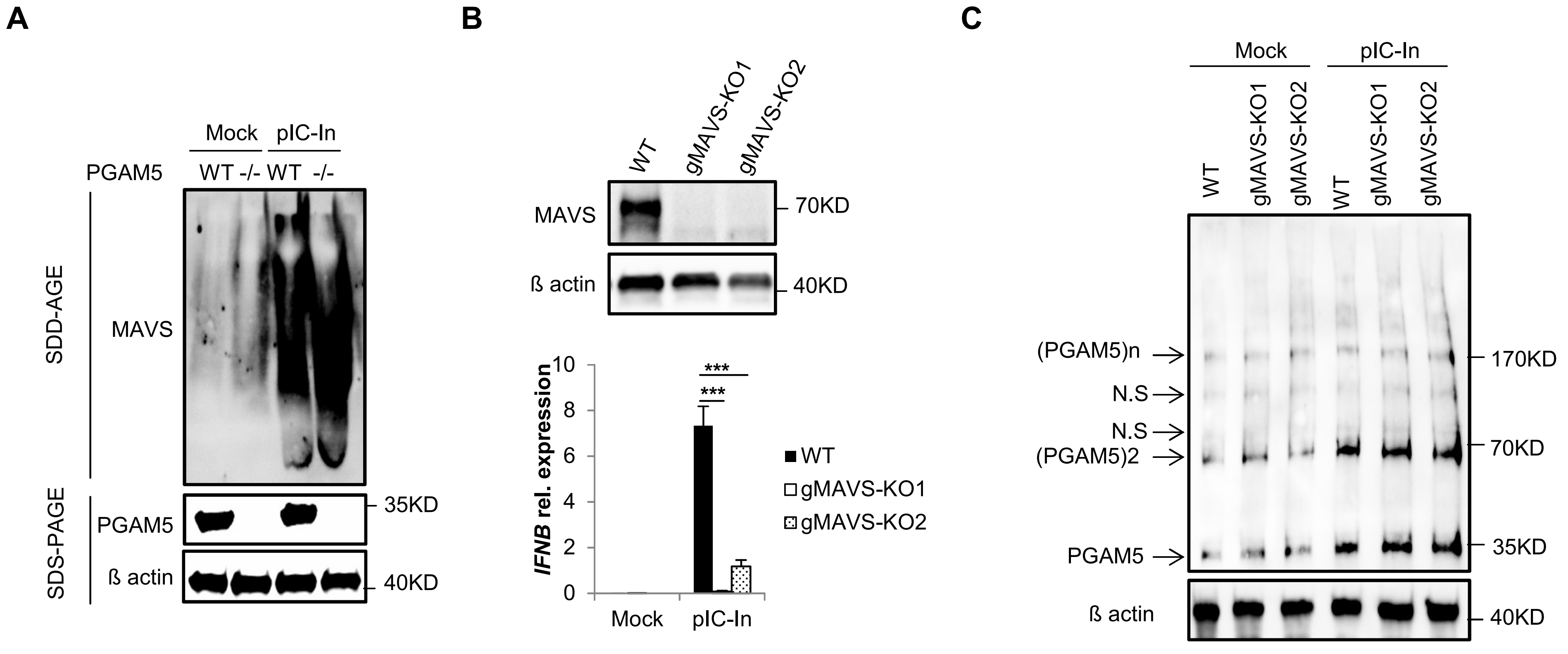
A
Mock
pIC-In
PGAM5
WT -/- WT -/-
SDD-AGE
MAVS
SDS-PAGE
PGAM5
ß actin
35KD
40KD
B
WT
gMAVS-KO1
gMAVS-KO2
MAVS
70KD
ß actin
40KD
IFNB rel. expression
10
8
6
4
2
0
***
***
Mock
pIC-In
WT
gMAVS-KO1
gMAVS-KO2
C
Mock
pIC-In
WT
gMAVS-KO1
gMAVS-KO2
WT
gMAVS-KO1
gMAVS-KO2
(PGAM5)n
N.S
N.S
(PGAM5)2
PGAM5
170KD
70KD
35KD
ß actin
40KD

S Figure 3

Figure 1

Fig 1B

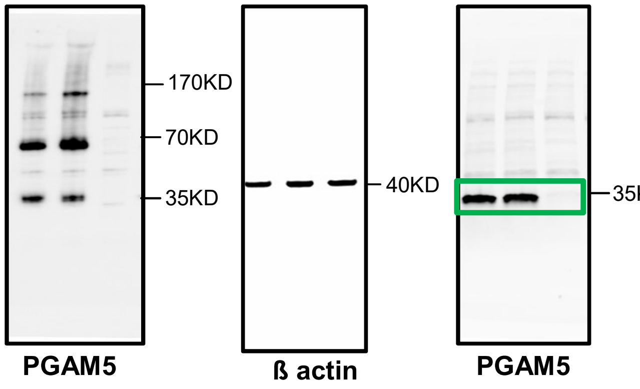

Figure 2

Fig 2A

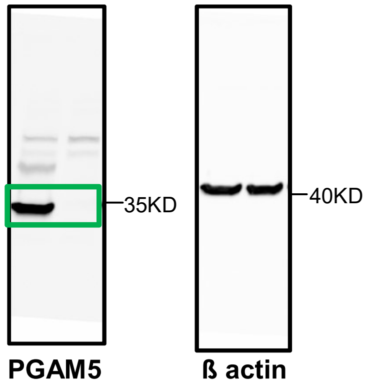

Fig 2D

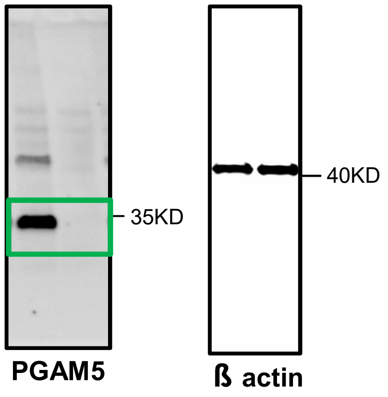

Fig 2F

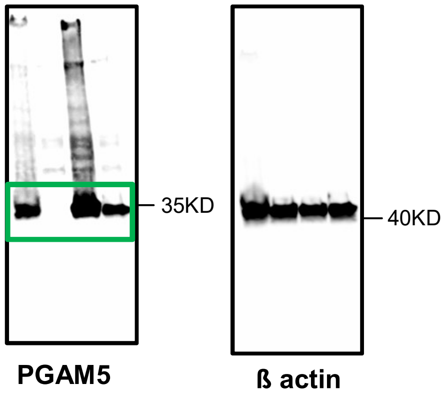

Figure 3

Fig 3A

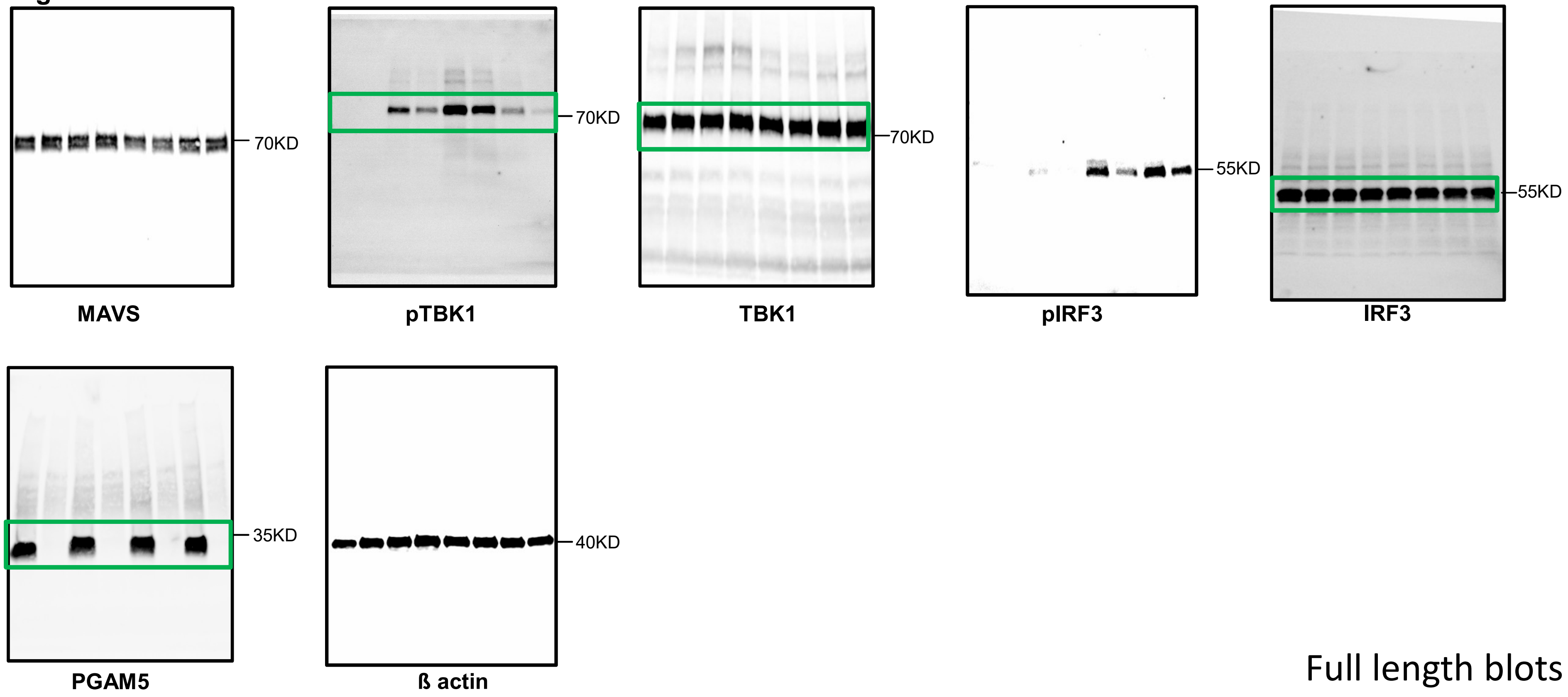

Full length blots

Figure 3

Fig 3D

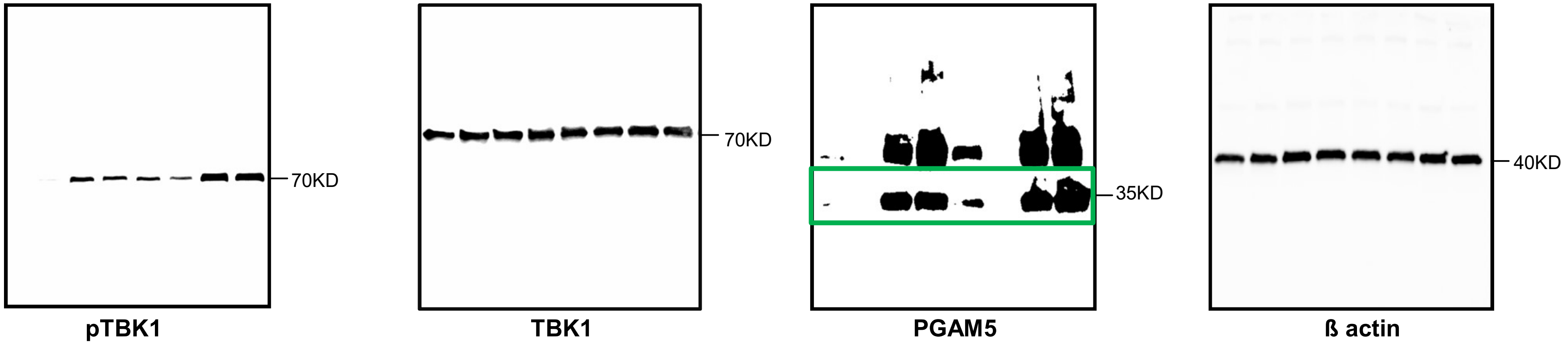

Fig 3F

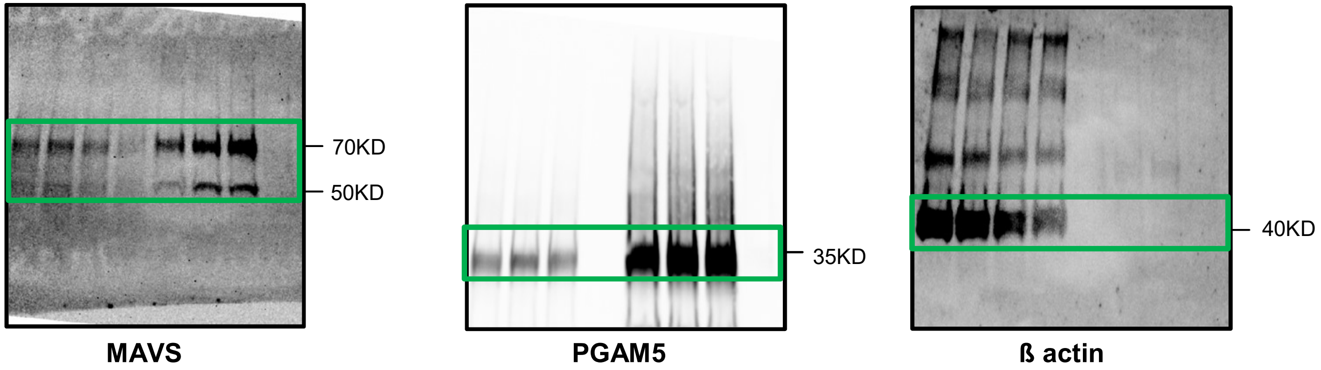

Figure 4

Fig 4A

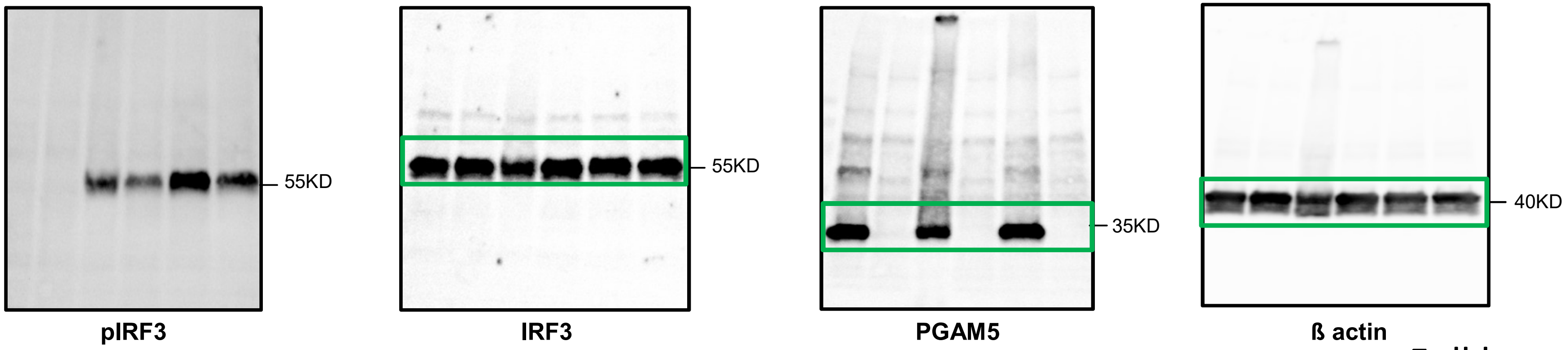

Full length blots

S Figure 1

S Fig 1A

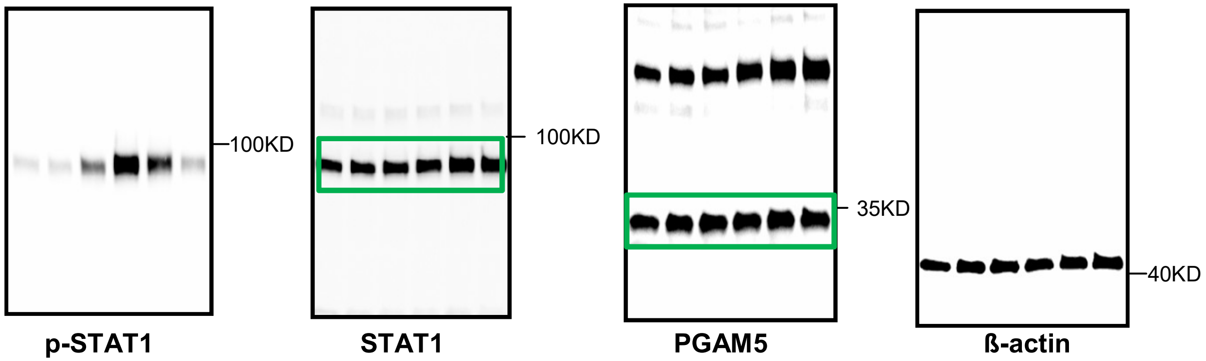

S Fig 1B

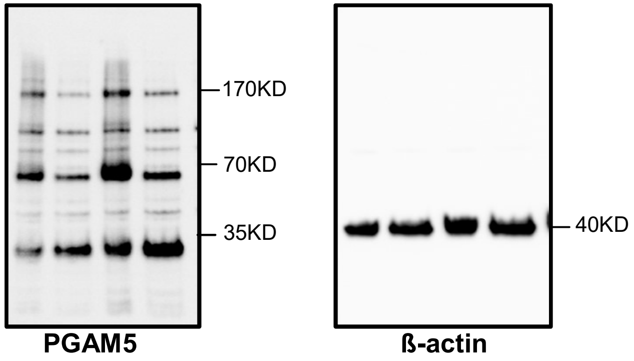

S Figure 2

S Fig 2C

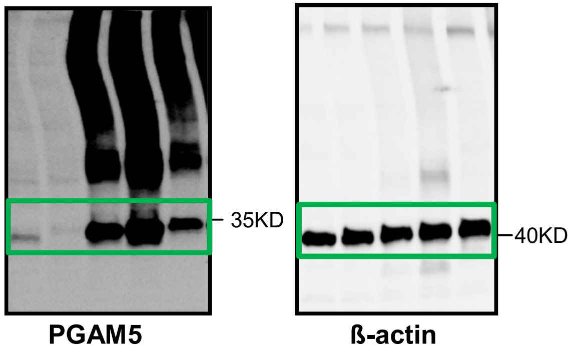

S Figure 3

S Fig 3A

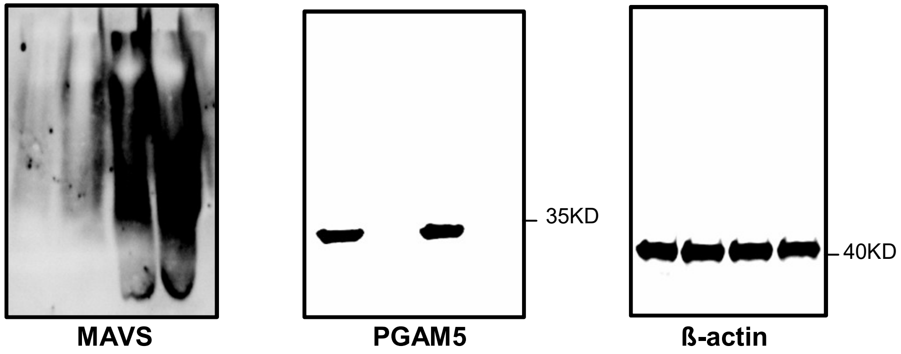

S Figure 3

S Fig 3B

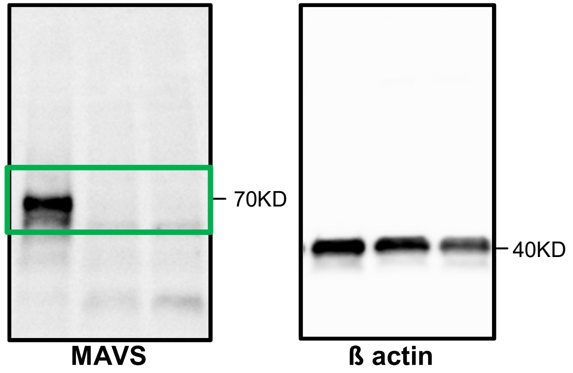

S Fig 3C

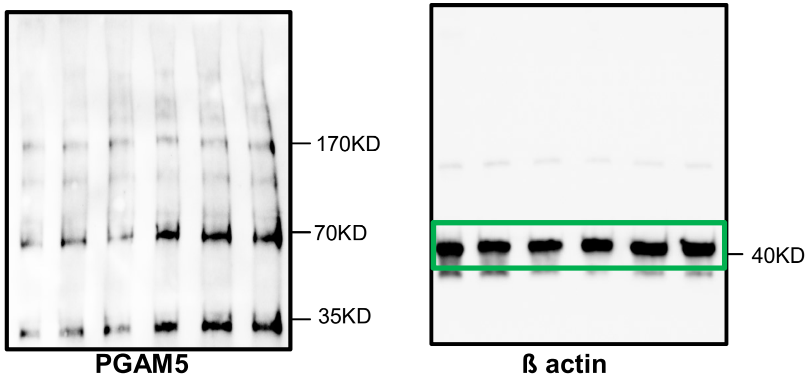

Full length blots
